# Supplementary material for: Identification of photoperiod-induced specific miRNAs in the adrenal glands of Sunite sheep (Ovis aries)
Source: Front Vet Sci. 2022 Jul 22;9:888207. doi: 10.3389/fvets.2022.888207 (PMC9354845; doi:10.3389/fvets.2022.888207)
Supplement: Supplementary file 1 [file Data_Sheet_1.ZIP › Supplementary file/miRDeep2 file.zip!/miRDeep2 file/Know_Structure/SPLP42A1/oar-mir-376a.pdf]

miRBase precursor : oar-mir-376a
Total read count : 149
oar-miR-376a-5p read count : 102
oar-miR-376a-3p read count : 45
remaining reads : 2

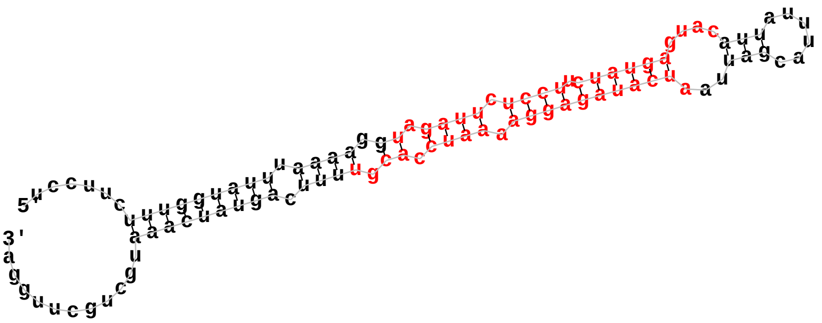

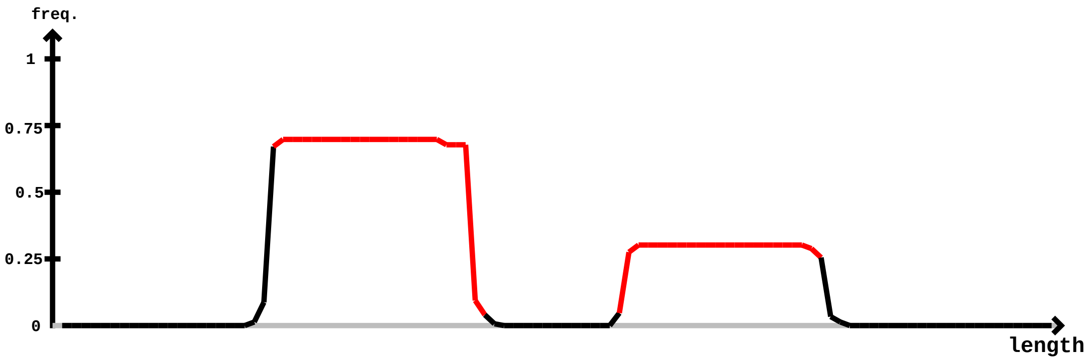

oar-miR-376a-5p

oar-miR-376a-3p

```
5'- uccuucuuugguauuuaaaaggugauucuccuucuaugaguacauuauuuacgauuaaucauagaggaaaauccacguuuucaguaucaaaugcugcuugga -3'    exp
    ......((((((((.((((.((.((((.((((.((((((....(((......)))...)))))))))).)))).)).)))).))))))))).............   reads   mm   sample
    ...................agguagauucuccuucuaugagu.............................................................   2       0    seq
    ....................gguagauucuccuucuaug................................................................   2       0    seq
    ....................gguagauucuccuucuaugagu..............................................................   9       0    seq
    .....................guagauucuccuucuaug................................................................   1       0    seq
    .....................guagauucuccuucuaugagu.............................................................   74      0    seq
    .....................guagauucuccuucuaugagua............................................................   6       0    seq
    .....................guagauucuccuucuaugaguac...........................................................   5       0    seq
    .....................guagauucuccuucuaugaguaca..........................................................   1       0    seq
    ......................uagauucuccuucuaugagu.............................................................   2       0    seq
    ......................uagauucuccuucuaugagua............................................................   2       0    seq
    .........................................................aaucauagaggaaaauccacgu........................   7       0    seq
    ..........................................................aucauagaggaaaauccac..........................   2       0    seq
    ..........................................................aucauagaggaaaauccacg.........................   5       0    seq
    ..........................................................aucauagaggaaaauccacgu........................   26      0    seq
    ..........................................................aucauagaggaaaauccacguu.......................   1       0    seq
    ...........................................................ucauagaggaaaauccacguu.......................   2       0    seq
    ...........................................................ucauagaggaaaauccacguuu......................   2       0    seq
```
